# Crystal structures of the PH domains from Lbc family of RhoGEFs bound to activated RhoA GTPase

**DOI:** 10.1016/j.dib.2018.01.024

**Published:** 2018-02-20

**Authors:** Zhe Chen, Steven Gutowski, Paul C. Sternweis

**Affiliations:** a Department of Biophysics, The University of Texas Southwestern Medical Center, 6001 Forest Park Road, Dallas, TX 75390, United States; b Department of Pharmacology, The University of Texas Southwestern Medical Center, 6001 Forest Park Road, Dallas, TX 75390, United States

## Abstract

The Pleckstrin homology (PH) domains from the Lbc family of Rho Guanine Nucleotide Exchange Factors (Lbc RhoGEFs) interact with activated Rho family GTPases. All 7 Lbc RhoGEFs associate directly with activated Rho GTPases via their PH domains. However, the binding affinities between the PH domains and the GTPases vary greatly. Here we present two crystal structures at resolutions of 1.4 Å and 2.0 Å of RhoA complexed with the PH domain from p114RhoGEF (PDB access code 6BCB) and AKAP-LbcRhoGEF (PDB access code 6BCA), respectively. These high resolution structures, together with the earlier structures of PDZRhoGEF-PH·RhoA and p190RhoGEF-PH·RhoA complexes, identify a highly conserved interface between the PH domains from Lbc-RhoGEFs and activated Rho GTPases. This manuscript is related to the manuscript titled “Direct Regulation of p190RhoGEF by Activated Rho and Rac GTPases” published in the Journal of Structural Biology.

**Specifications Table**

**Table:** 

Subject area	*Biology*
More specific subject area	*Regulation of Rho GTPase*
Type of data	*Table, text file, and figure*
How data was acquired	*X-ray crystallography*
Data format	*analyzed*
Experimental factors	*Proteins were expressed from bacterial host and purified with affinity tag and size-exclusion chromatography*
Experimental features	*Complex of proteins were purified and crystallized. Diffraction data from single crystals collected at synchrotron radiation source*
Data source location	*N/A*
Data accessibility	*Data described in this article has been deposited into the RCSB protein data bank, with access code of 6BCA (AKAP-LbcRhoGEF:RhoA) and 6BCB (p114RhoGEF:RhoA).*

**Value of the data**

• The Pleckstrin homology domains have long been associated with their binding to phospholipids. Direct interactions between PH domains and other proteins have been reported, however, structural insight into such interactions has been scarce.
• Crystal structures of the p114-PH and AKAP-Lbc-PH have not been reported before. Crystal structures of the PH:RhoA complexes will be of value to the scientific community focusing on Rho Guanine Nucleotide Exchange Factors in general. Structures reported here are of much higher resolution compared with the first complex published in 2010 between the PDZRhoGEF and activated RhoA.
• This is the first comprehensive analysis of binding affinities between all family members of the Lbc RhoGEFs and activated RhoA.

## 1 Data

### 1.1 Binding affinities measured by FRET between PH domains from the Lbc-RhoGEF family members and activated RhoA bound to GTPγS

**Fig. 1:**
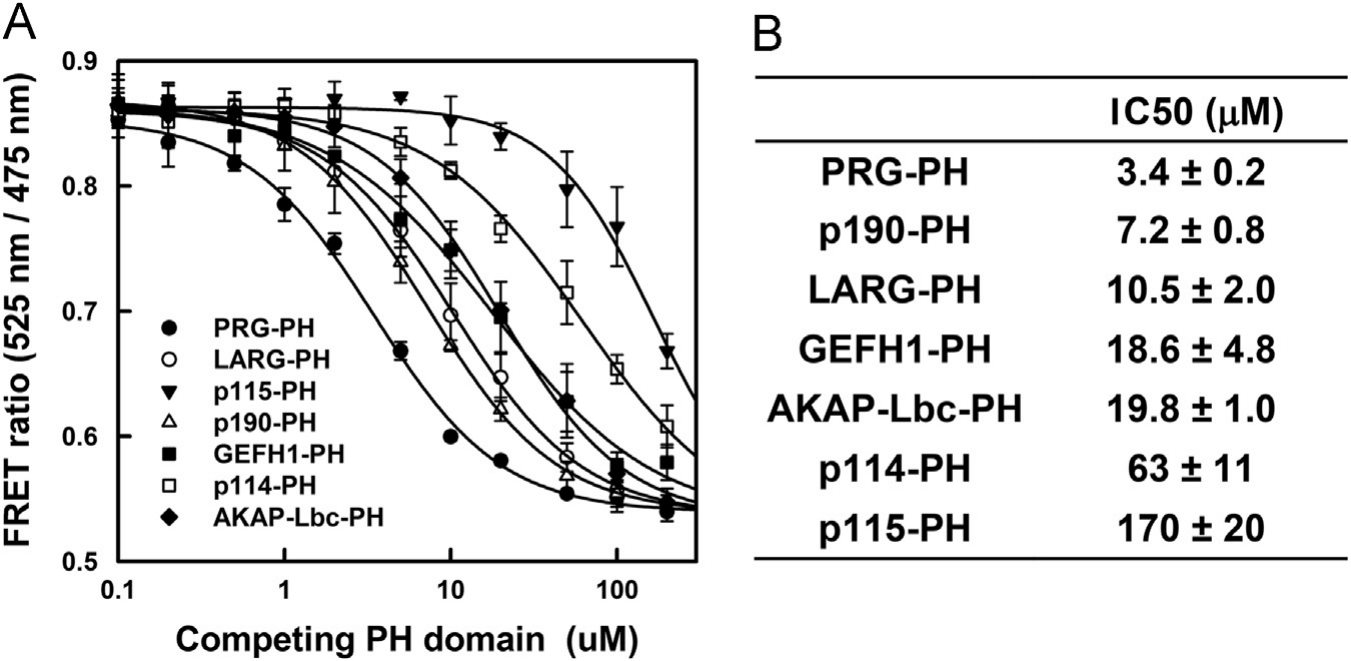
Binding affinities between PH domains of Lbc-RhoGEFs and activated RhoA measured by FRET. A. The ability of non-tagged PH domain to bind to RhoA·GTPγS was measured by competition of FRET produced by binding of 1 μM YFP-RhoA·GTPγS to 1 μM CFP-PRG-PH. Error bars represent the standard deviation of 3 independent experiments. B. The binding affinities, as indicated by the IC50 values, are summarized.

The binding affinities between PH domains from Lbc-RhoGEFs and RhoA·GTPγS were determined by titrating non-fluorescent PH domains into an equimolar mixture of YFP-RhoA·GTPγS and CFP-PRG-PH, and monitoring the decrease in intermolecular FRET (Fig. 1A). The binding affinity between the PH domain of PRG and RhoA·GTPγS has been reported [1], therefore allowing estimation of binding affinities between the other PH domains from Lbc-RhoGEFs and RhoA·GTPγS based on the comparative ability of the non-fluorescent PH domains to compete for binding with the fluorescent PRG-PH. Although all PH domains from Lbc-RhoGEFs bind activated RhoA [2], the binding affinities vary greatly, ranging from an IC_50_ of 3 μM for PRG-PH, to over 150 μM for p115-PH (Fig. 1B). Other PH domains, including those from p190RhoGEF, LARG, GEFH1 and AKAP-Lbc show relatively higher affinities with IC_50_s towards RhoA·GTPγS ranging between 10 and 20 μM, whereas p114-PH shows significantly lower affinity with an IC_50_ around 60 μM. The binding affinities between several PH domains from Lbc-RhoGEFs and RhoA·GTPγS have also been independently measured by isothermal titration calorimetry (ITC), with results similar to the values reported here [1].

### 1.2 Crystal structure of the p114RhoGEF-PH·RhoA·GTPγS complex

**Fig. 2:**
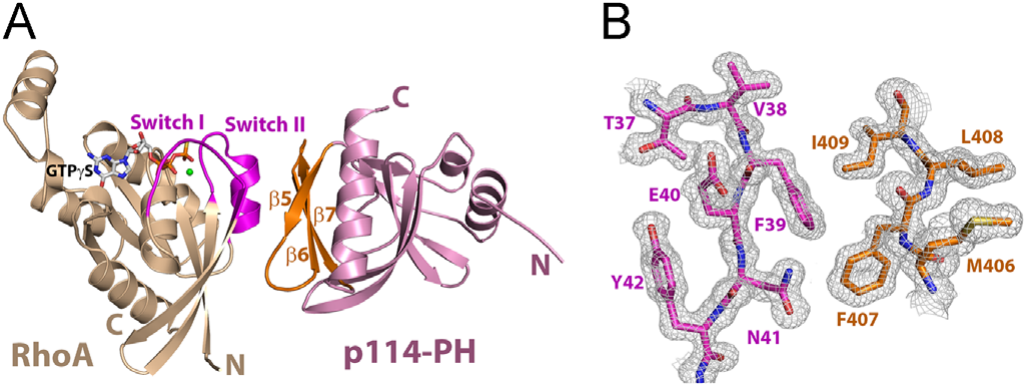
Structure of the p114 PH domain in complex with activated RhoA. A. Ribbon diagrams depicting tertiary structures of p114-PH in a complex with RhoA·GTPγS. p114-PH is colored light purple, with the C-terminal layer of β-strands colored orange. RhoA is colored wheat, with switch regions colored purple. GTPγS and magnesium ion are depicted as ball-and-stick models and colored as follows. Oxygen, nitrogen, carbon and phosphorous atoms are colored red, blue, grey, and yellow, respectively. Magnesium is colored green. B. Representative portion of the 1.4 Å electron density map (2*m*F_0_-*D*F_c_) contoured at σ = 1.0, showing part of the interface between activated RhoA and the PH domain of p114RhoGEF, using the same coloring scheme as in panel A.

The structure of RhoA·GTPγS bound to the PH domain of p114RhoGEF was determined at a resolution of 1.4 Å by molecular replacement using separate search models for a modified PRG-PH domain and RhoA (Fig. 2). The model was refined to conventional and free crystallographic *R* values of 13.6% and 16.5%, respectively. The final atomic model comprises residues 299–444 of p114-PH, and residues 1–181 of RhoA bound to GTPγS and Mg^2+^. An ethylene glycol molecule (from the cryo-protecting solution) is also observed in the structure. The remaining residues of the PH domain are disordered. Data collection and refinement statistics for the structure are summarized in Table 1. The electron density is well defined for GTPγS and Mg^2+^ in the guanine nucleotide binding pocket of RhoA. As observed in other Lbc-RhoGEF-PH·RhoA·GTPγS complex structures [1], [3], the activated RhoA binds directly to the C-terminal β-strands of the PH domain primarily via the switch I and switch II regions (Fig. 2A). The structure of the PH domain from p114 shares a similar fold to other PH domains in the Lbc RhoGEF family [1], [3], [4], [5], [6], [7], [8].

**Table 1:** Data collection and structure refinement statistics.

	**AKAP-Lbc-PH: RhoA**	**p114-PH: RhoA**
**Data collection**		
Source	APS SBC 19ID	APS SBC 19ID
Wavelength (Å)	0.9794	0.9794
Space group	P1	P2_1_
Unit cell (Å)		
*a, b, c* (Å)	51.35, 60.53, 63.62	48.93, 60.04, 65.48
*α, β, γ* (°)	91.83, 92.97, 90.01	90.00, 108.14, 90.00
Resolution (Å)	2.0	1.4
Rsym^*^	0.10 (0.40)	0.06 (0.56)
*I/σI*	11.0 (1.8)	23.5 (2.0)
Unique reflections	50,558 (2,327)	70,421 (3,332)
Completeness (%)	97.4 (90.7)	99.6 (95.1)
Redundancy	2.5 (1.9)	4.5 (3.4)
Wilson B-factor (Å^2^)	22.8	12.3
**Refinement**		
Resolution (Å)	34.2–2.0	27.3–1.4
No. reflections	50,552	70,380
*R*_*work*_*/R*_*free*_ (%)	19.6/23.4	13.6/16.5
Number of atoms	5,652	3,213
Protein	5,099	2,655
Ligand/ion	66	37
Water	487	431
Average B-factor (Å^2^)	24.0	20.0
rms deviations		
Bond lengths (Å)	0.015	0.010
Bond angles (°)	1.532	1.343
Ramachandran *favored/allowed/disallowed* (%)	96.8/3.2/0.0	98.8/1.2/0.0

^⁎^ Values in parentheses are for highest-resolution shell.

### 1.3 Crystal structure of the AKAP-LbcRhoGEF-PH·RhoA·GTPγS complex

**Fig. 3:**
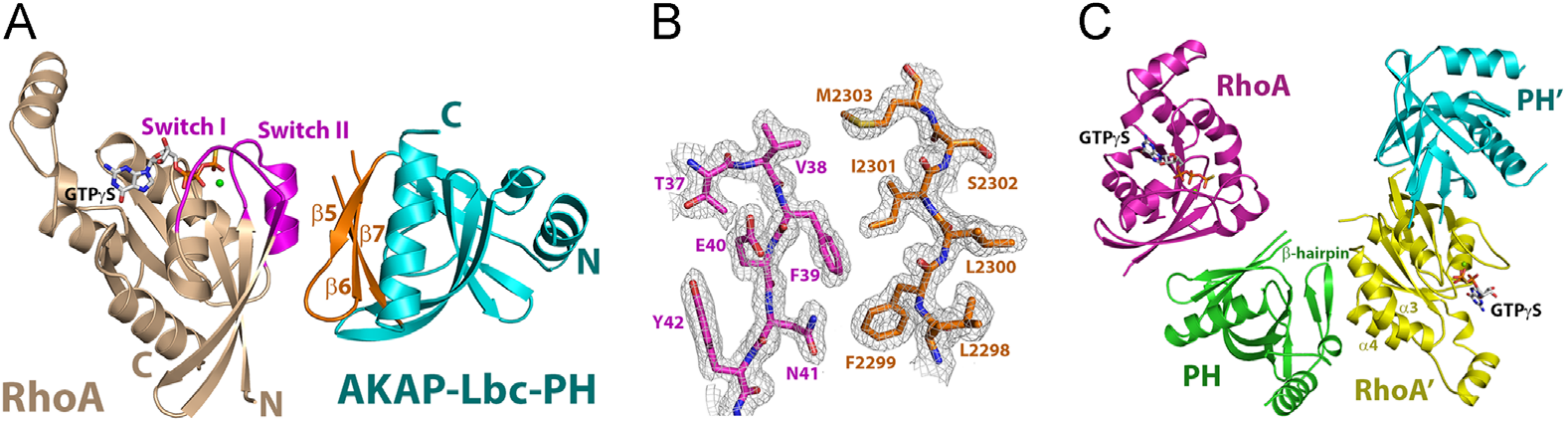
Structure of the AKAP-Lbc PH domain in complex with activated RhoA. A. Ribbon diagrams depicting tertiary structures of AKAP-Lbc-PH in a complex with RhoA·GTPγS. AKAP-Lbc-PH is colored blue, with the C-terminal layer of β-strands colored orange. RhoA and the bound GTPγS and magnesium ion are depicted and colored as in Fig. 2. B. Representative portion of the 2.0 Å electron density map (2*m*F_0_-*D*F_c_) contoured at σ = 1.0, showing part of the interface between activated RhoA and the PH domain of AKAP-LbcRhoGEF, using the same coloring scheme as in panel A. C. Ribbon diagrams depicting the two AKAP-Lbc-PH:RhoA complexes in the asymmetric unit of the crystal structure.

The structure of RhoA·GTPγS bound to the PH domain of AKAP-LbcRhoGEF was determined at a resolution of 2.0 Å by molecular replacement using separate search models for a modified PRG-PH domain and RhoA (Fig. 3). The model was refined to conventional and free crystallographic *R* values of 19.6% and 23.4%, respectively. There are two complexes in the asymmetric unit (Fig. 3C). The final atomic model comprises residues 2195–2304 and 2307–2333 of the AKAP-Lbc-PH domain, and residues 3–181 of RhoA bound to GTPγS and Mg^2+^. The remaining residues of the PH domain or RhoA are disordered. Data collection and refinement statistics are summarized in Table 1. The electron density is well defined for GTPγS and Mg^2+^ in the guanine nucleotide binding pocket of RhoA. Here again, the switch I and switch II regions of RhoA interacts directly with the β5, β6 and β7-strands of the PH domain from AKAP-Lbc (Fig. 3A). The structure of the PH domain from AKAP-Lbc shares a similar fold to other PH domains in the Lbc RhoGEF family. The two complexes in the asymmetric unit are held together by an interface formed between the α3 and α4 helices of RhoA, and the β-hairpin module near the N-terminal helix of AKAP-Lbc-PH that lies outside of the canonical core of the PH domain (Fig. 3C), and buries a surface area of about 330 A^2^. There is however no evidence that the complex of AKAP-Lbc-PH and RhoA forms a dimer based on size-exclusion chromatography.

### 1.4 The PH domain from p114RhoGEF presents a smaller binding surface towards activated RhoA compared with AKAP-LbcRhoGEF

The overall structures of the two PH·RhoA complexes reported here are very similar (Fig. 2, Fig. 3); residues from the PH domains that interact with activated RhoA are well conserved among all Lbc-RhoGEFs [1]. However, the PH domain from p114RhoGEF is less effective competing against PRG-PH in the FRET competition assay (Fig. 1), with an IC_50_ over 60 μM. AKAP-Lbc-PH on the other hand, shows stronger binding towards RhoA·GTPγS, with an IC_50_ value of about 20 μM. A closer examination of the two structures reveals that the interface between p114-PH and activated RhoA buries less surface area (approximately 500 A^2^) than that between AKAP-Lbc-PH and RhoA (about 570 A^2^). The difference mainly arises from the positioning of the C-terminal β-strands on PH relative to RhoA·GTPγS in the complex. When the two structures are superimposed based on coordinates from RhoA, the β5-β6 loop from p114-PH is farther away from the comparable RhoA interface in the complex with AKAP-Lbc-PH. Residues from the β5-β6 loop on PH participate in the interaction with RhoA·GTPγS [1], [3]. The reduced affinity between p114-PH and activated RhoA therefore could be explained by the subtle structural differences between individual PH domains.

## 2 Experimental design, materials and methods

### 2.1 Protein expression and purification

Coding regions of the PH domains from mouse p114RhoGEF (residues 302–444) and human AKAP-LbcRhoGEF (residues 2195–2333), as well as a C-terminally truncated fragment of RhoA (residues 1–181), were subcloned into a modified pGEX-KG vector containing the protease recognition site for the Tobacco Etch Virus (pGEX-KG-TEV), and expressed and purified from *E. coli* strain BL21(DE3) cells as described before [2], [3]. A 6His-tag was added to the C-terminus of p114-PH. Expression and purification of CFP-PH domains were carried out as described [1].

### 2.2 Formation and crystallization of PH:RhoA·GTPγS complexes

Exchanging of GTPγS for bound GDP on purified RhoA was carried out as described [3]. Equal moles of RhoA·GTPγS and the PH domain were mixed and then filtered through the gel filtration columns pre-equilibrated with a buffer containing 25 mM TrisCl, pH 8.5, 1 mM DTT, 100 mM NaCl and 1 mM EDTA and 2 mM MgCl_2_. Fractions that contained the PH·RhoA·GTPγS complex (molecular weight of approximately 40 kDa as judged by elution volume) were pooled and concentrated using Amicon-Ultra 4 (10 kDa) concentrators (Millipore) to a final concentration of 20 mg/ml. Aliquots (20 μl) of the concentrated complex were flash frozen with liquid nitrogen and stored at − 80 °C. All proteins were crystallized by vapor diffusion at 20 °C. The p114-PH·RhoA·GTPγS complex was crystallized from 22–24% polyethylene glycol 3350, 100 mM Bis-Tris, pH 5.8–6.1, and 200 mM ammonium sulfate. The AKAP-Lbc-PH·RhoA·GTPγS complex was crystallized from 24–26% polyethylene glycol 3350, 100 mM Bis-Tris, pH 5.5, and 200 mM ammonium sulfate. Crystals were then cryoprotected with an additional 15% (v/v) ethylene glycol.

### 2.3 Data collection and structure determination

Native data were measured at 100 K at the Structural Biology Center (Beamline 19ID) at Argonne National Laboratory. Diffraction data were reduced using the HKL software package [9]. Initial phases were generated by molecular replacement using the coordinates RhoA (PDB entry 1A2B) and a modified PRG-PH domain as separate search models, using program PHASER [10]. The coordinates of PRG-PH (PDB entry 3KZ1) was modified based on protein sequence alignment by Clustal W [11] using program Sculptor from the Phenix software package [12]. Model building was performed using the program Coot [13]. The model was refined using the Phenix software package [12]. Putative water molecules within hydrogen bonding distance of at least one protein atom or other water oxygen atoms and with refined B-factors < 100 Å^2^ were included in the model. MolProbity [14] indicates that over 96% of the residues fall in the most favorable regions of ϕ, ψ conformational space [15]. Coordinates have been deposited in the Protein Data Bank [16] with accession code 6BCB (p114-PH·RhoA) and 6BCA (AKAP-Lbc-PH·RhoA). Atomic representations were created using Pymol [17].

### 2.4 FRET competition assay (intermolecular FRET assay)

Intermolecular FRET was measured by association of 1 μM YFP-RhoA·GTPγS with 1 μM CFP-PRG-PH. Competition was measured by titration of non-fluorescent PH domains. Assays were done in 20 mM NaHEPES, pH 7.5, 1 mM EDTA, 1 mM DTT, 100 mM NaCl, 5 mM MgCl_2_ in a volume of 140 μl, utilizing a Fluorolog-3 fluorimeter at 25 °C at λ_ex_ = 433 nm, λ_em_ scanned from 450 to 560 nm, slits = 1/1 nm. Changes in FRET were reported as the ratio of fluorescence at 525 nm over 475 nm. Competition by each PH domain was measured three times. Error bars in titration curves show the standard deviation for averaged data points; reported IC_50_ values are the average of 3 independent determinations and variance is the standard deviation.

## Contributions

Z.C., S.G. and P.C.S. designed research, analyzed data and wrote the paper; Z.C., and S.G. performed research.
